# The impact of agricultural green finance on the level of agricultural green development

**DOI:** 10.1371/journal.pone.0323703

**Published:** 2025-06-05

**Authors:** Aihua Tong, Huawei Niu, Lili Jiang, Yifeng Wang

**Affiliations:** 1 School of Economics and Management, China University of Mining and Technology, Xuzhou, Jiangsu, China; 2 School of Economics and Management, Suqian University, Suqian Jiangsu, China; Huanggang Normal University, CHINA

## Abstract

Agricultural green finance is an important means to boost the development of agricultural green. Based on the panel data of 30 provinces in China from 2011 to 2021, this paper theoretically discusses the impact of agricultural green finance on the level of agricultural green development and conducts an empirical study by using the two-way fixed effect model and panel threshold model. The empirical findings show that agricultural green finance can significantly improve the level of agricultural green development mainly through facilitating the level of agricultural green technology innovation. The promoting effect of agricultural green finance on the level of agricultural green development is remarkable in the eastern region and non-major grain-producing areas, but not obvious in the central and western regions and major grain-producing areas. Indeed, the impact has a single threshold effect with environmental regulation as the threshold. Therefore, efforts should be made to improve the level of agricultural green development from the following aspects: vigorously developing agricultural green finance; promoting agricultural green technology innovation; strictly implementing environmental protection policies; and designing appropriate environmental regulation zones.

## Introduction

With the increasing awareness of ecological civilization and green development, great importance has been attached to promoting high-quality economic development in China. Agriculture is by no means insulated from green development in this context. Indeed, the green development of agriculture has become the main development direction of China’s agriculture. The *Opinions on Promoting Green Agricultural Development through Innovation Mechanisms* issued in 2017 called for accelerating green agricultural development. The 20th Report of the Communist Party of China and China’s “14th Five-Year Plan” also emphasized promoting the green transformation of agriculture.

Promoting the green development of agriculture not only requires a large amount of funds, but also poses an urgent need to transfer risks in the process of agricultural green development. Therefore, promoting the green development of agriculture cannot be separated from the support of finance, especially the strong support of agricultural finance and green finance. With the constant development of green finance, green financial products continue to penetrate into the agricultural field. Accordingly, the organic combination of green finance and agricultural finance gradually fosters agricultural green finance. As it is, agricultural green finance is the concrete embodiment of green finance in the agricultural field, mainly including agricultural green credit, agricultural insurance, green investment, and other green financial products. The impact of agricultural green finance on the level of agricultural green development and its impact mechanism is a topic worthy of attention. How can we scientifically measure the level of agricultural green development and the level of agricultural green finance development? What are the specific effects and mechanisms of agricultural green finance on the level of agricultural green development? The in-depth analysis of the above problems is of great practical significance and theoretical value for promoting agricultural green finance and agricultural green development.

Scholars have conducted relevant studies on the impact of green finance and rural finance on the green development of agriculture. For instance, Fanghui Pan, et al. (2024) [1] revealed that green finance plays a significant role in promoting the green development of agriculture. Based on the survey data of five provinces and cities, Junwei Zhang, et al. (2020) [2] analyzed the incentive effect of rural credit and agricultural insurance on the green development of agriculture. However, there is relatively little research on the impact of agricultural green finance on the level of agricultural green development. Does agricultural green finance have an impact on the level of agricultural green development? If it does, what is the specific impact mechanism? Based on the panel data of 30 provinces in China from 2011 to 2021, this paper theoretically discusses the impact of agricultural green finance on the level of agricultural green development, and conducts an empirical study by using the two-way fixed effects model and panel threshold model. The aim of this study is to find a path for agricultural green finance to effectively support agricultural green development and put forward some policy suggestions for agricultural green finance so as to better promote agricultural green development.

The marginal contribution of this paper mainly includes the following four aspects: Firstly, the relevant theories of agricultural green finance are further improved; and an evaluation index system is constructed with seven indicators selected from the four dimensions, namely, agricultural green credit, agricultural insurance, green investment, and policy support. This system can be used to measure the development level of agricultural green finance in 30 provinces in China from 2011 to 2021. Secondly, an evaluation index system of agricultural green development level is established with 17 indicators selected from five dimensions of resource conservation, environmental friendliness, ecological conservation, quality and efficiency improvement, and economic benefits. This system can be used to measure the agricultural green development level of 30 provinces in China from 2011 to 2021. Thirdly, the two-way fixed effect model is adopted to analyze the specific impact of agricultural green finance on the level of agricultural green development and the corresponding mechanism. Fourthly, environmental regulation factors are included in the research framework of the relationship between agricultural green finance and agricultural green development, and the panel threshold model is used to analyze whether environmental regulation has a threshold effect in the process of agricultural green finance and agricultural green development.

## Literature review

### Research on agricultural green development

The relevant theories about agricultural green development can be traced back to the sustainable development theory. The concept of agricultural sustainable development first appeared in *Building a Sustainable Society* by Brown (1981) [3]. Harlem and Brundtland (1987) [4] emphasized “sustainable development” in *Our Common Future*. Haggblade and Hazell (1989) [5] pointed out that the difficulty and core of agricultural green development consists in the innovation of agricultural green development path and green technology. Pimentel, et al. (2005) [6] believed that agricultural green development is a development mode based on ecological agriculture. Unep (2011) [7] proposed that agricultural green development includes resource conservation, good ecological environment, and long-term coordinated development of agriculture. Parviz Koohafkan et al. (2012) [8] observed that agricultural green development would improve the safety and quality of agricultural products and enhance the brand impact of agricultural products. Zhaohai Bai et al. (2018) [9] proposed that agricultural green development should be the green development of the whole agricultural industry chain. Adnan et al. (2019) [10] analyzed the main influencing factors of agricultural green development, including individual characteristics of farmers, family characteristics, farmers’ cognition, and external environment characteristics. According to Qi Wei et al. (2018) [11], agricultural green development is a process of investigating sustainable development based on the principles of honoring the environment and utilizing a range of contemporary technologies. Yujing Zhang and Fei Ye (2022) [12] pointed out that agricultural green development is an environmentally friendly model of sustainable agricultural development.

The construction of an evaluation index system for agricultural green development level has not been unified. To be specific, evaluation index systems of agricultural green development have been constructed from the following dimensions: resource conservation, environmental friendliness, ecological conservation and quality and efficiency [11, 13]; supply capacity, resource utilization, environmental quality, ecological maintenance, and farmers’ life [14]; resource utilization, environmental impact, ecological conservation, and economic benefits [15].

### Research on agricultural green finance

Guangwen He (2016) [16] pointed out that rural green finance can be understood as a rural financial development strategy, by which financial institutions can promote the implementation of sustainable agricultural development strategy through the operation of financial businesses. Jiujie Ma et al. (2017) [17] proposed that agricultural green finance refers to the effective promotion and implementation of sustainable agricultural development by the financial sector through environmentally friendly financial services. Baichuan Cheng (2018) [18] maintained that agricultural green finance means guiding social funds to agricultural green development through financial instruments such as loans, equity investments, bonds, insurance, and stocks.

### Research on the impact of finance on agricultural green development

So far, quite little has been done to explore the impact of agricultural green finance on agricultural green development. Yet, some relevant research has been conducted to reveal the impact of finance and green finance on agricultural green development.

Research on the impact of finance on agricultural green development. Mingxian Li and Hui Bai (2019) [19] explored the necessity of credit to support agricultural green development. Lei Wang and Jinming Ma (2023) [20] concluded that digital inclusive finance can significantly improve the level of agricultural green development. Aihua Tong and Lili Jiang(2022) [21] empirically maintained that agricultural credit has a significant positive impact on promoting agricultural green development in Jiangsu Province. Dejiang Chu and Yan Zou. (2023) [22] proposed that financial instruments are an important means for the government to promote agricultural green development.

Research on the impact of green finance on agricultural green development. Jun Ma et al. (2021) [23] proposed that agriculture is deeply affected by environment and climate and should be one of the key fields supported by green finance. Jie Pang, et al. (2022) [24] claimed that green financial products such as green credit, green insurance, green bonds, green funds, and carbon finance have a wide range of application scenarios in promoting agricultural green development. Gujie Li et al. (2022) [25] discovered that green finance can significantly improve agricultural green total factor productivity. The research results of Yalin Mo et al. (2023) [26] indicate that the development of green finance can significantly reduce China’s agricultural carbon emission intensity, which is conducive to promoting agricultural green development. According to Xiaojun Xiao and Mingqi Hu (2023) [27], green finance not only has local effect in promoting agricultural green development but exerts some significant positive spatial spillover effect as well.

### Research gap

Existing studies mainly focus on the connotation and measurement of agricultural green development and the connotation of agricultural green finance. In other words, quite little has been done to measure the development level of agricultural green finance and evaluate the impact of agricultural green finance on agricultural green development. In this case, in-depth study should be conducted to evaluate the specific impact of agricultural green finance on agricultural green development and reveal its impact mechanism. Based on the theoretical analysis, this paper empirically analyzes the specific impact of agricultural green finance on agricultural green development and its impact mechanism by using the two-way fixed effects model and panel threshold model.

## Theoretical analysis and hypothesis

### Direct impact of agricultural green finance on the level of agricultural green development

#### Agricultural green credit is conducive to providing funds for the green development of agriculture.

Through differentiated credit policies, agricultural green credit optimizes the allocation of credit resources, improves the efficiency of fund use and allocation, and guides more funds to flow to areas conducive to the green development of agriculture. First of all, banks and other financial institutions increase financial support for energy-saving and environmental protection enterprises through green credit. This is conducive to the development of these enterprises, thus optimizing the ecological environment for the green development of agriculture. Secondly, agricultural green credit can provide financial support for agricultural business entities by adopting agricultural green technologies and methods. Mingyue Li and Kai Chen (2020) [28] observed that during the transition period of agriculture to high-quality green development, the increase in capital accumulation of agricultural operators will increase their willingness to adopt agricultural green production methods and thus improve the level of agricultural green development. In addition, agricultural green credit is conducive to enhancing the agricultural administrative subjects’ awareness of green development and prompting them to consciously abide by the green system. In this way, the agricultural production factors can be combined rationally to reduce the use intensity of agricultural inputs that produce pollution in the process of agricultural production, thus promoting the green development of agriculture.

#### Agricultural insurance is conducive to coping with the risks in the process of agricultural green development.

Agricultural insurance has the functions of risk diversification and loss compensation, which is conducive to coping with various risks, such as natural risks and market risks, in the process of agricultural green development. In this sense, it can provide risk protection for agricultural green development. Through innovation, insurance institutions have introduced agricultural environmental liability insurance, planting and breeding weather insurance, agricultural product quality and safety assurance insurance, and other types of insurance, by which most of the risks faced by agricultural operators in the process of agricultural green development can be effectively dealt with. Accordingly, agricultural insurance is conducive to promoting agricultural green development.

#### Green investment is conducive to optimizing the ecological environment for agricultural green development.

Promoting the green development of agriculture requires a good agricultural ecological environment such as air, soil, and water. To optimize the agro-ecological environment demands a large amount of capital investment. By increasing the capital input for improving the environment, green investment such as investment in urban environmental infrastructure management, investment in industrial pollution control, and fiscal expenditure for environmental protection contributes remarkably to the optimization of the ecological environment for agricultural green development. Accordingly, it can be assumed that green investment is highly conducive to promoting the green development of agriculture.

#### Policy signal effect.

Under the background of the “double carbon” goal to reach the peak of carbon neutrality and building a green financial system from top to bottom, the green finance policy, as a national macro-financial regulation policy, conveys the signal of vigorously promoting the development of the green economy through green finance to the outside world. The implementation of green finance policy in the agricultural field is conducive to promoting the development of agricultural green finance. To be specific, agricultural green finance helps to guide the transformation of traditional agricultural operators into green and low-carbon agricultural operators. In this way, green finance policy can greatly boost the green development of agriculture.

**Fig 1:**
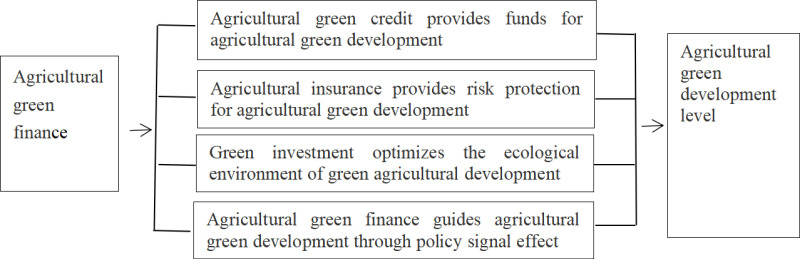
The impact of agricultural green finance on the level of agricultural green development.

To sum up, agricultural green credit provides financial support for promoting agricultural green development by playing the function of fund allocation, improving the efficiency of fund use and allocation. Agricultural insurance can provide risk protection for promoting the green development of agriculture. Green investment is conducive to optimizing the ecological environment for the green development of agriculture. Agricultural green finance guides agricultural green development through policy signal effect(**Fig 1**). Therefore, hypothesis H1 is proposed: agricultural green finance can promote the green development of agriculture.

### Impact mechanism of agricultural green finance on the level of agricultural green development

Agricultural green finance can improve the level of agricultural green development by influencing the innovation of agricultural green technology. Agricultural green finance can provide funds for agriculture-related technology research and development departments, thus promoting agricultural green technology innovation and boost agricultural technology progress. As more and more attention is paid to the green development of agriculture, agricultural operators need more agricultural green technology innovation to improve the efficiency of agricultural input factors and reduce the waste rate of agricultural resources. As the demand for agricultural green technology innovation increases in the agricultural production sector, the profitable opportunities for agricultural technology research and development increase. Qiuwang Cheng et al. (2022) [29] held that with the temptation of profit, R&D departments tend to carry out agricultural green technology research and development to maximize long-term profits. Agricultural green technology innovation cannot be separated from the support of agricultural green finance. Agricultural green credit can expand the financial scale and enrich the financing sources and channels of agricultural R&D departments. Agricultural insurance can provide risk protection for agricultural green technology innovation, thus facilitating its progress. Agricultural green technology innovation is not only conducive to improving the agricultural ecological environment but also to the adoption of green production methods by agricultural operators. In this sense, it promotes the green development of agriculture. Therefore, hypothesis H2 is proposed: agricultural green finance can improve the level of agricultural green development through the path of agricultural green technology innovation.

### Threshold effect of agricultural green finance on the level of agricultural green development

Jianpeng Zhang and Shiyi Chen (2021) [30] found that the synergistic effect of financial development and environmental regulation can greatly accelerate the green transformation of the economy. This paper includes environmental regulation factors into the research framework of the relationship between agricultural green finance and agricultural green development level to explore whether this relationship is non-linear with the change of environmental regulation intensity. Longzheng Du et al. (2019) [31] maintained that environmental regulation has a threshold effect, which plays different roles before and after the turning point. Neng Shen and Fengchao Liu (2012) [32] proposed that when the intensity of environmental regulation exceeds a specific threshold, its promotion effect on technological innovation will be more significant. In the field of agriculture, agricultural green finance can promote the green transformation of agricultural development mode by facilitating agricultural green technology innovation. The impact of agricultural green finance on agricultural green development will be affected by the intensity of environmental regulations. Accordingly, environmental regulation may have threshold effect in the impact of agricultural green finance on agricultural green development level. Different environmental regulation intensities will affect the functioning of agricultural green technology innovation. With the increase of environmental regulation intensity, agricultural green technology innovation comes to function gradually. Therefore, hypothesis H3 is proposed: environmental regulation intensity plays a threshold effect in the impact of agricultural green finance on the level of agricultural green development.

## Research design

### Variable selection

#### Explained variable.

The explained variable is the level of agricultural green development. With reference to the existing relevant studies, combined with the connotation and characteristics of agricultural green development and the availability of data, 17 indicators were selected to construct the evaluation index system of agricultural green development level from five dimensions: resource conservation, environmental friendliness, ecological conservation, quality and efficiency improvement, and economic benefit, as shown in **Table 1**. The entropy method was used to determine the indicators weight and measure the agricultural green development index. The agricultural green development index ranges from 0 to 1, and the closer it is to 1, the higher the level of green agricultural development.

**Table 1:** Evaluation index system of agricultural green development level.

Dimension Index	Specific indicators	Unit of measurement	Index measurement	Attributes
**Resource conservation**	Multi-cropping index of arable land	–	Crop sown area/arable land area	negative
	Water consumption per gross agricultural output value	m^3^	Agricultural water consumption/gross agricultural output value	negative
	Proportion of water-saving irrigation area	%	Agricultural water-saving irrigation area/agricultural irrigation area	positive
	Agricultural diesel oil application intensity	kg/ha	Agricultural diesel oil application quantity/sown area	negative
**Environmental friendliness**	Fertilizer application intensity	kg/ha	Fertilizer application quantity/sown area	negative
	Pesticide application intensity	kg/ha	Pesticide application quantity/sown area	negative
	Agricultural film application intensity	kg/ha	Quantity of agricultural film used/sown area	negative
**Ecological conservation**	Forest coverage rate	%	China Environmental Statistical Yearbook	positive
	Proportion of soil erosion control area	%	Soil erosion control area/provincial area	positive
	Proportion of crop disaster area	%	Crop disaster area/crop sown area	negative
**Quality and efficiency improvement**	Number of green food products per sown area	Piece/10,000 hectares	Number of green food products/sown area	positive
	Number of green food enterprises per sown area	Piece/10,000 hectares	Number of green food enterprises/sown area	positive
	Total agricultural output value per sown area	RMB yuan 10,000/ha	Gross agricultural output value/crop sown area	positive
	Per capita gross output value of primary industry	RMB yuan 10,000/ha	Gross output value of primary industry/Number of employees in primary industry	positive
	Grain yield per unit area	kg/ha	Grain production/grain sown area	positive
**Economic benefit**	Per capita disposable income of rural residents	RMB yuan	China Rural Statistical yearbook	positive
	Agricultural GDP per capita	RMB yuan 10,000/person	Value added of primary industry/rural population	positive

In this paper, the entropy method was used to determine the indicators weight in that this method can help obtain a more objective weight. The specific steps of entropy method are as follows.

The first step is to standardize the data. The original data of all indicators are standardized by the dispersion standardization method, and 0.0001 is added to the standardized formula to avoid the meaningless assignment of zero values. There are n provinces, k years, and j indicators. The positive index data are standardized by formula (1), and the negative index data by formula (2).

Positive indicator normalization formula:


$$x_{ikj}^{\prime }=\frac{x_{ikj}-min(x_{kj})}{max(x_{ij})-min(x_{ij})}+0.0001\;$$ (1)

Negative indicator normalization formula:


$$x_{ikj}^{\prime }=\frac{max(x_{ij})-x_{ikj}}{max(x_{ij})-min(x_{ij})}+0.0001\;$$ (2)

where, $x_{ikj}^{\prime }\;$ denotes the indicator data after standardization; $x_{ikj}$ represents the original data of the j-th indicator of province i in the k-th year; $min(x_{ij}$ denotes the minimum value of the j-th indicator; and $max(x_{ij})$ represents the maximum value of the j-th indicator.

The second step is to determine the weight of the k-th sample value under the j-th indicator:


$$\begin{array}{c} {\text{p}}_{ikj}=\frac{x_{ikj}^{\prime }}{\;\sum_{\text{k = 1}}^{\text{K}}\sum_{\text{i = 1}}^{\text{n}}x_{ikj}^{\prime }\;}\; \end{array}$$ (3)

The third step is to calculate the entropy value of the j-th indicator:


$$e_{j}=-r\sum_{k=1}^{K}\sum_{i=1}^{n}p_{ikj}\ln\left(p_{ikj}\right)\;$$ (4)

Where   r=ln(n

The fourth step is to calculate the entropy redundancy of the j-th indicator:


$$\begin{array}{c} {\text{d}}_{j}=1-e_{j\;}\; \end{array}$$ (5)

The fifth step is to calculate the weight of each indicator:


$$\begin{array}{c} {\text{w}}_{j}=\frac{d_{j}}{\sum_{j=1}^{m}d_{j}}\; \end{array}$$ (6)

The sixth step is to calculate the comprehensive score:


$$\begin{array}{c} {\text{s}}_{ik}=\sum_{j=1}^{m}{\text{w}}_{j}x_{ikj}^{\prime }\; \end{array}$$ (7)

#### Core explanatory variable.

The core explanatory variable is the development level of agricultural green finance. Based on the connotation of agricultural green finance and considering the availability of data, this paper selects 7 indicators from four dimensions: agricultural green credit, agricultural insurance, green investment, and policy support to build an evaluation index system of agricultural green finance development level, as shown in **Table 2**. The entropy method was used to measure the development level of agricultural green finance.

**Table 2:** Evaluation index system of agricultural green finance development level.

Dimension Index	Specific indicators	Unit of measurement	Index measurement	Attributes
**Agricultural green credit**	Loans for agriculture, forestry, animal husbandry and fishery	Hundred million RMB yuan	China Financial Statistical Yearbook	positive
	Percentage of interest expenses in six high energy-consuming industries	%	Interest expenses of six high energy-consuming enterprises/total industrial interest expenses	negative
**Agricultural insurance**	Agricultural insurance density	RMB yuan	Agricultural premiums/primary industry population	positive
	Agricultural insurance depth	%	Agricultural premium/Added value of agriculture, forestry, animal husbandry and fishery	positive
**Green investment**	Proportion of investment in urban environmental pollution control	%	Urban environmental pollution control investment/GDP	positive
	Proportion of investment in industrial pollution control	%	Industrial pollution control investment/GDP	positive
**Policy support**	Percentage of environmental protection expenditure	%	Fiscal expenditure on environmental protection/total fiscal expenditure	positive

#### Control variables.

In fact, the level of agricultural green development is also affected by a variety of factors. In order to study the impact of agricultural green finance on the level of agricultural green development more comprehensively, it is necessary to add control variables that may affect the level of agricultural green development. With reference to existing literature, five variables, namely rural human capital level (edu), rural resident income level (lnrni), rural household fixed asset investment (lnfa), fiscal expenditure to support agriculture (fis), and imports and exports of agricultural products (lnainex), were selected as control variables.

①Rural human capital level (edu). The level of rural human capital affects the input of agricultural factors. The improvement of rural human capital level is conducive to the adoption of agricultural green production mode and agricultural green development. In this paper, the average years of education (edu) of rural residents is used to represent the level of rural human capital.

②Rural resident income level. The increase in the income level of rural residents will help increase the input of green agricultural materials, adopt green agricultural production methods, and thus improve the level of agricultural green development. In this paper, the logarithm of real per capita disposable income of rural residents (lnrni) is used to represent the income level of rural residents.

③Investment in fixed assets of rural households. The increase in fixed asset investment of rural households is also conducive to the adoption of green production methods and technologies by agricultural management entities, thus improving the level of agricultural green development. In this paper, the logarithm of farmers’ investment in fixed assets (lnfa) is used to represent investment in fixed assets of rural households.

④Fiscal expenditure to support agriculture. The increase in fiscal support for agriculture can definitely help promote agricultural development. However, it does not necessarily contribute to green development of agriculture, which mainly depends on whether the fiscal support for agriculture is invested in the field of agricultural green development. Therefore, the impact of fiscal support expenditure on the level of agricultural green development embodies some kind of uncertainty. In this paper, the proportion of agricultural, forestry, and water affairs expenditures to the general budget expenditures (fis) is used to represent fiscal expenditure to support agriculture.

⑤Imports and exports of agricultural products. The import of agricultural products represents the opening-up degree of agriculture. Generally speaking, the improvement of agricultural opening to the outside world is conducive to the import of agricultural green production technology and helps to improve the level of agricultural green development. In this paper, the logarithm of the sum of imports and exports of agricultural products of each province (lnainex) is used to represent the imports and exports of agricultural products.

#### Intermediary variable.

In order to explore the impact mechanism of agricultural green finance on the level of agricultural green development, agricultural green technology innovation was selected as the mechanism variable and represented by the number of agricultural green patent applications.

#### Threshold variable.

Based on the above theoretical analysis, environmental regulation (envi) was selected as the threshold variable. Drawing on the research of Lei Wang, et al. (2023) [20], the threshold variable is represented by the proportion of local fiscal environmental protection expenditure to the gross agricultural output value.

### Data sources

The data used in this paper are panel data of 30 provinces (municipalities and districts) in China (excluding Tibet and Hong Kong, Macao, and Taiwan regions) from 2011 to 2021, mainly from *China Rural Statistical Yearbook*, *China Industrial Statistics Yearbook*, the website of the National Bureau of Statistics, the website of the National Intellectual Property Office, and the Wind database. It should be noted that the per capita disposable income of rural residents is deflated using the corresponding index based on 2011 to eliminate the impact of inflation and price fluctuations. In order to unify the dimensions and alleviate the multi-collinearity, the scale variables, such as per capita disposable income of rural residents, fixed asset investment of rural households, and imports and exports of agricultural products, are taken in logarithmic form. For the missing values of some variables in individual years, the linear interpolation method was used to fill in the gap.

### Descriptive statistics of variables

The descriptive statistics of each variable are shown in **Table 3**. As can be seen, the mean value of agricultural green development level is 0.4118, the maximum value is 0.6260, and the minimum value is 0.2591, indicating that the agricultural green development level varies greatly among different provinces. The mean value of agricultural green finance development level is 0.2898, with the maximum value and the minimum value being 0.5662 and 0.1920, respectively. This indicates that great differences exist in the development level of agricultural green finance among different provinces.

**Table 3:** Descriptive statistics of variables.

Variable	Variable name	Mean value	Standard deviation	Minimum value	Maximum value
**agd**	Agricultural green development level	0.4118	0.6766	0.2591	0.6260
**agf**	Agricultural green finance development level	0.2898	0.0558	0.1920	0.5662
**edu**	Average years of schooling for rural residents	7.7522	0.5898	5.8476	9.6603
**lnrni**	Real per capita disposable income of rural residents	9.2946	0.3988	8.2091	10.5385
**lnfa**	Rural household fixed asset investment	5.3700	1.1343	0.7419	6.8739
**fis**	Fiscal expenditure to support agriculture	0.1142	0.0332	0.0411	0.2038
**lnainex**	Import and export of agricultural products	12.4194	1.6656	7.6046	15.2710
**pagp**	Number of agricultural green patent applications	0.8211	2.0203	0.0082	16.1679
**envi**	Environmental regulation	0.1932	0.4329	0.0224	3.5311

### Model construction

#### Benchmark regression model.

Based on the above internal logical relationship and theoretical basis between agricultural green finance and agricultural green development level, a benchmark regression model (8) is established to further empirically test the impact of agricultural green finance on agricultural green development level and verify the hypothesis H1 proposed in this paper. The regression model is shown as follows:


$${agd}_{it}=\beta_{0}+\beta_{1}{agf}_{it}+\sum_{j=2}^{6}\beta_{j}X_{it}+\sigma_{i}+\mu_{t}+\varepsilon_{it}\;$$ (8)

Where i represents the region; t denotes the time; ${agd}_{it}\;$ is the agricultural green development level of province i in t year; ${agf}_{it}\;$ refers to the development level of agricultural green finance of the province in t year; β is the parameter to be estimated; $X_{it}\;$ denotes a series of control variables, including rural human capital level (edu), rural resident income level (lnrni), rural household fixed asset investment (lnfa), fiscal expenditure to support agriculture (fis), and imports and exports of agricultural products (lnainex); $\sigma_{i}$ is the fixed effect of provinces; $\mu_{t}$ is the year fixed effect; and $\varepsilon_{it}$ represents the random disturbance term.

#### Intermediary effect model.

In order to test the impact mechanism of agricultural green finance on the level of agricultural green development, drawing on the practice of Shiyi Chen and Dengke Chen (2018) [33], this paper firstly empirically analyzes the impact of agricultural green finance development on the level of agricultural green development, then explores the impact of agricultural green finance development on the institutional variable agricultural green technology innovation. Moreover, this paper also analyzes the impact of institutional variable agricultural green technology innovation on the level of agricultural green development. On the basis of baseline regression model (8), impact mechanism models (9) and (10) are set up to verify the afore-said hypothesis H2.


$${pagp}_{it}=\gamma_{0}+\gamma_{1}{agf}_{it}+\sum_{j=2}^{6}\gamma_{j}X_{it}+\sigma_{i}+\mu_{t}+\varepsilon_{it}\;$$ (9)

$${agd}_{it}=\tau_{0}+\tau_{1}p{agp}_{it}+\sum_{j=2}^{6}\tau_{j}X_{it}+\sigma_{i}+\mu_{t}+\varepsilon_{it}\;$$ (10)

Where pagp denotes the number of agricultural green patent applications per capita, representing agricultural green technology innovation and functioning as an intermediary variable. Other variables can be interpreted in the same way as formula (8). Formula (9) tests the relationship between the development level of agricultural green finance and the intermediary variable of agricultural green technology innovation; Formula (10) tests the relationship between the intermediary variable agricultural green technology innovation and agricultural green development level.

#### Panel threshold model.

In order to further study the relationship between agricultural green finance and agricultural green development level, environmental regulation is taken as the threshold variable. Using the threshold model of [Hansen, 1999] [34] as reference, the panel threshold model (11) is constructed:


$$\begin{array}{cc} {agd}_{it} & =\beta_{0}+\beta_{11}{agf}_{it}\times I\left(envi\leq \rho_{1}\right)+\beta_{12}{agf}_{it}\times I\left(\rho_{1}<\rho envi\leq \rho_{2}\right)+\cdots +\beta_{1n}{agf}_{it}\\ & \;\times I\left(\rho_{n-1}<envi\leq \rho_{n}\right)+\beta_{1(n+1)}{agf}_{it}\times I\left(envi>\rho_{n}\right)+\sum_{j=2}^{6}\beta_{j}X_{it}+\sigma_{i}+\varepsilon_{it} \end{array}$$ (11)

Where I (∙) denotes the indicative function, being 1 if the corresponding condition is true, and 0 if it is not; envi represents environmental regulation; $\rho_{1},\cdots ,\rho_{n}$ is the threshold value, and the interpretation of other variables is the same as that of formula (8).

## Analysis of empirical results

### Changing trend of agricultural green development level

**Fig 2:**
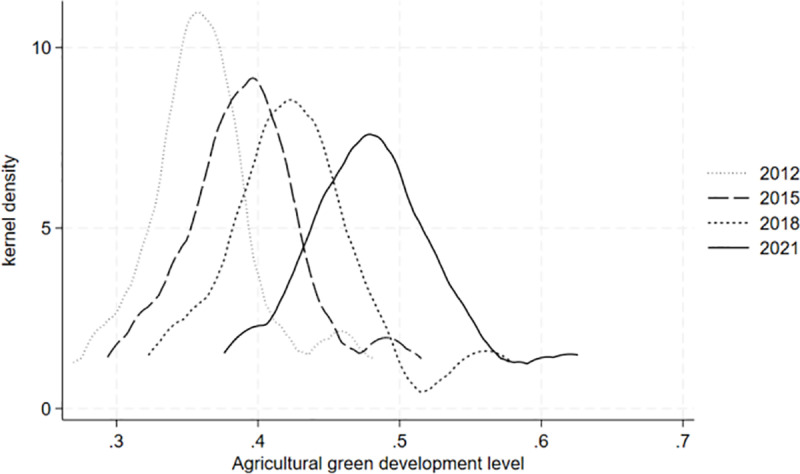
Kernel density of agricultural green development level.

**Fig 2** displays a kernel density estimate of the agricultural green development level of 30 provinces in China, which is conducted in 2012, 2015, 2018, and 2021 as time nodes. From the perspective of the evolution trend, the center of the distribution curve gradually shifts to the right, indicating that the overall level of agricultural green development is constantly improving. In terms of the distribution pattern, the height of the main peak experiences a “gradual decline” evolution process, and the width of the main peak experiences a “slow increase” evolution process, indicating that the differences in the levels of agricultural green development among various provinces in China witness an expanding trend. From the perspective of extensibility, there is a certain right-tailing phenomenon, indicating that some provinces in China have a higher level of agricultural green development, and the distance from the average level has expanded. In general, the level of agricultural green development in 30 provinces in China from 2011 to 2021 exhibits an overall trend of improvement. This indicates that some green production measures taken by China in the agricultural field in recent years, such as reducing the use of agricultural materials such as fertilizers, pesticides, agricultural films, and other measures to optimize the agricultural ecological environment, have achieved certain results.

### Changing trend of agricultural green finance development level

**Fig 3:**
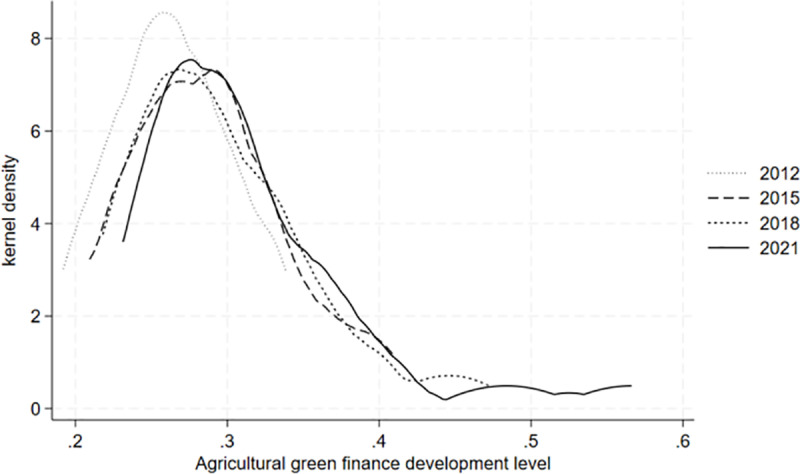
Kernel density of agricultural green finance development level.

**Fig 3** displays a kernel density estimate of the development level of agricultural green finance in 30 provinces of China, which is conducted in 2012, 2015, 2018, and 2021 as time nodes. From the perspective of the evolution trend, the center of the distribution curve moves first to the right, then to the left, and then slowly back to the right, indicating that the development level of agricultural green finance shows a trend of “improvement - decline - slow improvement” on the whole. As for the distribution pattern, the height of the main peak experiences a “rapid decline and slow rise” evolution process, and the width of the main peak experiences a “slow decrease” evolution process. This indicates that the difference in the development levels of agricultural green finance in various provinces in China shows a narrowing trend. From the perspective of extensibility, there is a relatively obvious right-tailing phenomenon, indicating that the development level of agricultural green finance in some provinces in China is relatively high, and the distance from the average level has expanded. In general, the development level of agricultural green finance in 30 provinces of China from 2011 to 2021 shows a certain volatility.

### Baseline regression results and analysis

Based on the above theoretical analysis and the panel data of 30 provinces in China from 2011 to 2021, this paper uses the two-way fixed effect model to test the impact of agricultural green finance on the level of agricultural green development so as to verify the theoretical hypothesis H1. Based on model (1), the estimation results of the impact effect of agricultural green finance on agricultural green development level after controlling individual effect and time-point effect are reported in **Table 4**. The test results of the relationship between agricultural green finance and agricultural green development level without adding control variables are shown in column (1) of **Table 4**. The estimated coefficient of agricultural green finance is significantly positive, indicating that agricultural green finance can significantly improve the level of agricultural green development. In **Table 4**, columns (2) to (5) present the test results of adding control variables gradually into the regression model, and the estimated coefficients of agricultural green finance are significantly positive. Column (6) of **Table 4** is the regression result after adding control variables, showing that the estimated coefficient of agricultural green finance is 0.1484, which is smaller than the regression result of column (1) in **Table 4**, 0.1663. This indicates that the impact of agricultural green finance on the level of agricultural green development would be overestimated and exaggerated when the impact of other control variables is not taken into account. Accordingly, the research hypothesis H1 is validated. The promotion effect of agricultural green finance on the level of agricultural green development has gradually become prominent, and agricultural green finance has become an indispensable force to improve the level of agricultural green development.

**Table 4:** Baseline regression results of the impact of agricultural green finance on the level of agricultural green development.

Variable	(1)	(2)	(3)	(4)	(5)	(6)
	agd	agd	agd	agd	agd	agd
**agf**	0.1663^**^	0.1563^**^	0.1734^**^	0.1542^***^	0.1510^***^	0.1484^***^
	(0.0726)	(0.0654)	(0.0673)	(0.0477)	(0.0494)	(0.0505)
**edu**		0.0164^**^	0.0163^***^	0.0142^**^	0.0139^**^	0.0138^**^
		(0.0060)	(0.0058)	(0.0056)	(0.0055)	(0.0054)
**lnrni**			0.0409^***^	0.0393^***^	0.0389^***^	0.0374^***^
			(0.0091)	(0.0117)	(0.0115)	(0.0114)
**lnfa**				0.0163^*^	0.0168^*^	0.0159^*^
				(0.0087)	(0.0088)	(0.0088)
**czzn**					−0.0833	−0.0810
					(0.0955)	(0.0941)
**lnainex**						0.0032
						(0.0044)
**Constant**	0.3143^***^	0.1928^***^	−0.1737^*^	−0.2245^*^	−0.2115	−0.2314^*^
	(0.0193)	(0.0515)	(0.0982)	(0.1249)	(0.1251)	(0.1334)
**Provincial fixed effect** **Year fixed effect**	YES YES	YES YES	YES YES	YES YES	YES YES	YES YES
**Sample**	330	330	330	330	330	330
**Adjusted *R*** ^**2**^	0.9149	0.9180	0.9221	0.9273	0.9275	0.9275

**Note:** Standard error in parentheses.

*** ,

** , and

* represent significance levels at 1%, 5%, and 10%, respectively. The following tables are the same.

### Endogeneity analysis and robustness test

#### Endogeneity analysis.

Due to the possibility of a two-way causal relationship between agricultural green finance and agricultural green development level, measurement errors of indicators, and missing variables in the model, endogenous problems may be caused. In order to overcome the endogenous problem, this paper uses the instrumental variable model to estimate the impact of agricultural green finance on the level of agricultural green development again. According to Ruicai Yuan (2023) [35], (1) the core explanatory variable lags by one period; (2) both the core explanatory variables and the control variables lag by one period; (3) the lag of the development level of agricultural green finance (L.agf) is taken as an instrumental variable. On the one hand, the lag period of agricultural green finance is highly correlated with the current period of agricultural green finance, which accords with the hypothesis of the correlation of instrumental variables. On the other hand, the lag of agricultural green finance will not change significantly with the level of agricultural green development in the current period, which is in line with the hypothesis of exogenous instrumental variables.

The endogeneity test results are shown in **Table 5**. Column (1) of **Table 5** shows the regression results of the lag of the development level of agricultural green finance. Column (2) of **Table 5** displays the regression results for which both core explanatory variables and control variables lag by one period. Column 3) of **Table 5** shows the regression results of the instrumental variable method. The Kleibergen-Paap rk F statistic is 104.7870 and the Kleibergen-Paap rk LM statistic is 40.8300, indicating that the selected tool variables are reasonable. All of them pass the significance test, indicating that after the endogenous problem is identified, agricultural green finance still exerts a significant positive impact on the level of agricultural green development, further verifying the rationality of hypothesis H1. At the same time, after comparing the baseline regression results with the regression results of instrumental variables, it is found that ignoring the endogenous problem would underestimate the promoting effect of agricultural green finance on the level of agricultural green development.

**Table 5:** Results of endogeneity test.

Variable	(1)	(2)	(3)
	agd	agd	agd
**L.agf**	0.1397***	0.1316***	
	(0.0490)	(0.0474)	
**agf**			0.2090***
			(0.0527)
**Constant**	−0.2160*	0.2667	
	(0.1138)	(0.2326)	
**Control variable**	YES	YES	YES
**Provincial fixed effect** **Year fixed effect**	YES YES	YES YES	YES YES
**Kleibergen-Paap Wald rk F**			104.7870
**Kleibergen-Paap rk LM**			40.8300
**Sample**	300	300	300
Adjusted *R*^2^	0.9308	0.9307	0.1007

#### Robustness test.

The following robustness tests are performed in this paper. First, after excluding the sample data of four municipalities, the regression results are shown in column (1) of Table 6. Second, the core explanatory variable is replaced by the second-order lag of the development level of agricultural green finance (L2.agf), and the regression results are shown in column (2) of Table 6. Third, the LSDV method is adopted, and the regression results are shown in column (3) of Table 6. Fourth, all variables involved in the model are truncated at 1% and 99% quantiles, and the regression results are shown in column (4) of Table 6. All of them have passed the significance test, which verifies the robustness of the results in this paper.

**Table 6:** Robustness test results.

Variable	(1)	(2)	(3)	(4)
	agd	agd	agd	agd
**agf**	0.0877*		0.1484***	0.1484***
	(0.0457)		(0.0275)	(0.0505)
**L2.agf**		0.1096**		
		(0.0525)		
**Constant**	−0.1633	−0.3229**	−0.1646	−0.2314*
	(0.1230)	(0.1395)	(0.1078)	(0.1334)
**Control variable**	YES	YES	YES	YES
**Provincial fixed effect** **Year fixed effect**	YES YES	YES YES	YES YES	YES YES
**Sample**	286	270	330	330
**Adjusted *R*** ^**2**^	0.9443	0.9243	0.9676	0.9275

### Heterogeneity analysis

#### Heterogeneity analysis of eastern, central, and western regions.

There are great differences in agricultural resource endowment and economic development level in different regions of China. The level of agricultural green development is different in different regions, so is the level of agricultural green finance development. Therefore, the promotion effect of agricultural green finance on the level of agricultural green development may present regional heterogeneity. Based on this, this paper divides 30 provinces in China into three regions: eastern, central, and western regions, to test the heterogeneity of the impact of agricultural green finance on the level of agricultural green development. Column (1) of **Table 7** shows the regression result of the eastern region, which has passed the significance test, indicating that agricultural green finance can significantly improve the level of agricultural green development in the eastern region. Column (2) of **Table 7** presents the regression result of the central region, and column (3) of **Table 7** is the regression result of the western region, which fails to pass the significance test, indicating that agricultural green finance cannot significantly improve the level of agricultural green development in the central and western regions.

**Table 7:** Results of regional heterogeneity test.

Variable	(1) Eastern region	(2) Central region	(3) Western region
	agd	agd	agd
**agf**	0.1316***	0.0537	−0.0095
	(0.0357)	(0.0738)	(0.0617)
**Constant**	−2.0476**	−0.0985	0.1987
	(0.7289)	(0.2213)	(0.4838)
**Control variable**	YES	YES	YES
**Provincial fixed effect** **Year fixed effect**	YES YES	YES YES	YES YES
**Sample**	121	88	121
**Adjusted *R*** ^**2**^	0.9335	0.9556	0.9600

One possible reason is that the eastern region, due to its natural geographical location and climatic conditions, has a more developed agricultural economy, a better foundation for green agriculture, and a higher level of agricultural green development. At the same time, the eastern region has a faster economic development and a more developed financial market. Compared with that of the central and western regions, the financial system in the eastern region is more complete, and financial products are more diverse. The development level of agricultural green finance in the eastern region is higher. Therefore, when promoting agricultural green finance, the eastern region can adapt to the transformation more quickly. This, in turn, would render better effects in improving the level of agricultural green development in the eastern region than in the central and western regions.

#### Analysis of heterogeneity between major grain-producing areas and non-major grain-producing areas.

In this paper, 30 provinces in China were divided into major grain-producing areas and non-major grain-producing areas to test the heterogeneity of the impact of agricultural green finance on agricultural green development. Column (1) of **Table 8** displays the regression results of major grain-producing areas, which fail to pass the significance test, indicating that agricultural green finance could not significantly improve the level of agricultural green development in major grain-producing areas. Column (2) of **Table 8** shows the regression result of non-major grain-producing areas, which has passed the significance test, indicating that the development level of agricultural green finance can significantly improve the level of agricultural green development in non-major grain-producing areas.

**Table 8:** Test results of grain-producing area heterogeneity.

Variable	(1) Major grain-producing areas	(2) Non-major grain-producing areas
	agd	agd
**agf**	0.0623	0.1352**
	(0.0370)	(0.0633)
**Constant**	0.2875	−0.1801
	(0.4572)	(0.1785)
**Control variable**	YES	YES
**Provincial fixed effect** **Year fixed effect**	YES YES	YES YES
**Sample**	143	187
**Adjusted *R*** ^**2**^	0.9593	0.9295

The possible reasons mainly fall into two aspects. First, as the major grain-producing areas are the main source of grain supply in China, agricultural production activities in these areas are frequent, with relatively high intensity of use of agricultural inputs such as fertilizers and pesticides. This leads to greater obstacles for agricultural green finance in improving the level of agricultural green development. Second, it is related to the relatively high development level of agricultural green finance in non-grain-producing areas. Non-grain-producing areas include economically developed provinces and municipalities such as Beijing, Shanghai, and Guangdong. Compared with grain-producing areas, the financial systems in non-grain-producing areas are more complete, and financial practitioners are more experienced, resulting in a higher level of development of agricultural green finance.

### Mechanism test

The benchmark regression results verify that agricultural green finance can significantly improve the level of agricultural green development. It is still necessary to further explore and verify the impact path of agricultural green finance on agricultural green development so as to clarify the impact mechanism of agricultural green finance.

**Table 9** displays the test results of the impact mechanism of agricultural green finance on the level of agricultural green development. Column (1) of **Table 9** shows that agricultural green finance has a significant promoting effect on agricultural green technology innovation, and column (2) of **Table 9** shows that agricultural green technology innovation has a significant promoting effect on the level of agricultural green development, which verifies hypothesis H2.

**Table 9:** Results of the impact mechanism of agricultural green finance on the level of agricultural green development.

Variable	(1)	(2)
	pagp	agd
**agf**	14.8988**	
	(6.2192)	
**pagp**		0.0083**
		(0.0035)
**Constant**	3.2404	−0.2456*
	(11.0411)	(0.1294)
**Control variable**	YES	YES
**Provincial fixed effect** **Year fixed effect**	YES YES	YES YES
**Sample**	330	330
**Adjusted *R*** ^**2**^	0.5139	0.9367

### Threshold effect test

In this paper, the bootstrap method was used to sample 300 times to test the situation of environmental regulation as a threshold variable. The results are shown in **Table 10**. The P value corresponding to the single threshold is 0.000, and that corresponding to the double threshold is 1.000, indicating that there is a single threshold effect, and the single threshold value is 0.9169. The P value corresponding to a double threshold is 1.000, indicating the double threshold does not pass the significance test.

**Table 10:** Test results of threshold effect.

Threshold variable	Threshold type	Threshold value	F	P	1% critical value	5% critical value	10% critical value
**Envi**	Single threshold	0.9169	36.3700	0.000	29.7576	24.8576	20.4600
	Double threshold		0.1700	1.000	85.7371	63.3685	49.3845

The estimated results of single-panel threshold regression are shown in **Table 11**. As the regression results reveal, when the intensity of environmental regulation is at a low level (envi≤0.9169), the coefficient of impact of agricultural green finance on agricultural green development level is 0.0879. When the environmental regulation intensity exceeds the threshold value of 0.9169, the coefficient of impact of agricultural green finance on the level of agricultural green development increases to 0.2156. As it is, with the improvement of environmental regulation intensity, the impact of agricultural green finance on the level of agricultural green development presents a significant positive non-linear feature with increasing marginal effect. In other words, environmental regulation plays a threshold effect in the impact of agricultural green finance on agricultural green development, thus verifying hypothesis H3. Based on the above analysis, it is necessary to give full play of the synergistic effect of agricultural green finance and environmental regulation to promote agricultural green development. The government needs to make greater efforts to implement environmental protection policies, design appropriate environmental regulation zones, and optimize the ecological environment for agricultural green development.

**Table 11:** Threshold regression results.

Variable	agd
**agf × I(env**i ≤ **0.9169)**	0.0879**
	(0.0439)
**agf × I(envi > 0.9169)**	0.2156***
	(0.0365)
**Constant**	−0.9419
	(0.0595)
**Control variable**	YES
**Provincial fixed effect**	YES
**Year fixed effect**	YES
**Sample**	330
**Adjusted *R*** ^**2**^	0.8610

## Conclusions and policy recommendations

### Main conclusions

This paper theoretically discusses the impact of agricultural green finance on the level of agricultural green development. Based on the panel data of 30 provinces in China from 2011 to 2021, this paper empirically tests the impact of agricultural green finance on the level of agricultural green development by using the two-way fixed effect model and panel threshold model. The main conclusions are as follows: (1) Agricultural green finance can significantly improve the level of agricultural green development, and the conclusion is still valid after a series of robustness tests. (2) The impact of agricultural green finance on the level of agricultural green development exhibits certain heterogeneity. The promoting effect of agricultural green finance on the level of agricultural green development is more remarkable in the eastern region and non-grain-producing areas, but not obvious in the central and western regions and major grain-producing areas. (3) Agricultural green finance mainly improves the level of agricultural green development through the path of agricultural green technology innovation. (4) Threshold effect results show that the impact of agricultural green finance on the level of agricultural green development has a single threshold effect with environmental regulation as the threshold. Once exceeding the threshold of environmental regulation, agricultural green finance exerts a greater promoting effect on improving the level of agricultural green development.

### Policy recommendations

This paper provides new evidence for better interpreting the effect of agricultural green finance on the level of agricultural green development. As for how to improve the level of agricultural green development through agricultural green finance, this paper puts forward the following suggestions: (1) Vigorously developing agricultural green finance. To be specific, the top-level design of agricultural green finance should be optimized, while prioritizing the coordination and cooperation of agricultural green credit, agricultural insurance, green investment, and financial support. In this way, the institutional foundation for promoting the development of agricultural green finance can be consolidated. Meanwhile, on the basis of vigorously developing agricultural credit and green credit, the government departments should guide banks to actively design and promote agricultural green credit products that can more effectively meet the capital demands for the green development of agriculture. More efforts should be made to further improve the policy-based agricultural insurance system. The government departments should guide property insurance companies to actively develop and promote agricultural insurance products such as agricultural environmental liability insurance, agricultural meteorological insurance, and agricultural product quality and safety guarantee insurance, so as to give full play to agricultural green finance in promoting the level of green development of agriculture. (2) Vigorously promoting agricultural green technology innovation. Agricultural green technology innovation is the main intermediary variable of agricultural green finance, affecting the level of agricultural green development. In the field of agricultural technology innovation, more attention should be paid to agricultural green technology innovation. To be specific, more supporting funds should be given, and more agricultural scientific and technological personnel should be guided to agricultural green technology innovation. (3) Implementing environmental protection policies and designing appropriate environmental regulation zones. In order to better play the role of agricultural green finance in promoting the level of agricultural green development, it is necessary for government departments to strictly implement environmental protection policies, design appropriate environmental regulation intervals, and give full play to the synergistic effect of agricultural green finance and environmental regulation.

## Supporting information

S1 Data Basic data.
(XLSX)
